# Effect of pregnancy versus postpartum maternal isoniazid preventive therapy on infant growth in HIV-exposed uninfected infants: a post-hoc analysis of the TB APPRISE trial

**DOI:** 10.1016/j.eclinm.2023.101912

**Published:** 2023-03-17

**Authors:** Ashenafi S. Cherkos, Sylvia M. LaCourse, Daniel A. Enquobahrie, Barbra A. Richardson, Sarah Bradford, Grace Montepiedra, Blandina T. Mmbaga, Tapiwa Mbengeranwa, Gaerolwe Masheto, Patrick Jean–Phillippe, Nahida Chakhtoura, Gerhard Theron, Adriana Weinberg, Haseena Cassim, Mpho S. Raesi, Elsie Jean, Deo Wabwire, Teacler Nematadzira, Lynda Stranix-Chibanda, Anneke C. Hesseling, Linda Aurpibul, Amita Gupta, Grace John-Stewart, IMPAACT P1078 TB APPRISE Study Team, Timothy R. Sterling, Renee Browning, Katie McCarthy, Lisa Aaron, Katherine Shin, Amanda Golner, Bonnie Zimmer, Jyoti S. Mathad, Savita Pahwa, Vandana Kulkarni, Diane Costello, Vivian Rexroad, Monica Gandhi, Joan Du Plessis, Amy James Loftis

**Affiliations:** a Biostatistics and Epidemiology Department, School of Public Health, University of North Texas Health Science Center, Fort Worth, TX, USA; b Department of Epidemiology, School of Public Health, University of Washington, Seattle, WA, USA; c Department of Medicine, Division of Allergy and Infectious Diseases, University of Washington, Seattle, WA, USA; d Department of Global Health, School of Public Health, University of Washington, Seattle, WA, USA; e Department of Biostatistics, School of Public Health, University of Washington, Seattle, WA, USA; f FHI 360, Durham, NC, USA; g Center for Biostatistics in AIDS Research, Harvard T.H. Chan School of Public Health, Boston, MA, USA; h Department of Biostatistics, Harvard T.H. Chan School of Public Health, Boston, MA, USA; i Kilimanjaro Clinical Research Institute -Kilimanjaro Christian Medical Centre and Kilimanjaro Christian Medical University College, Moshi, Tanzania; j University of Zimbabwe College of Health Sciences-Clinical Trials Research Centre, Harare, Zimbabwe; k Botswana Harvard AIDS Institute Partnership, Gaborone, Botswana; l National Institute of Allergy and Infectious Diseases, Bethesda, MD, USA; m NIH, Eunice Kennedy Shriver National Institute of Child Health and Human Development (NICHD, Bethesda, MD, USA; n Department of Obstetrics and Gynaecology, Faculty of Medicine and Health Sciences, Stellenbosch University, Cape Town, South Africa; o Departments of Pediatrics, Medicine and Pathology, University of Colorado School of Medicine Anschutz Medical Campus, Aurora, CO, USA; p Perinatal HIV Research Unit, University of the Witwatersrand, South Africa; q Department of Pediatrics, GHESKIO Centers, Port-au-Prince, Haiti; r Makerere University – Johns Hopkins University Research Collaboration, Kampala, Uganda; s University of Zimbabwe Clinical Trials Research Centre, Harare, Zimbabwe; t Department of Paediatrics and Child Health, College of Health Sciences, University of Zimbabwe, Harare, Zimbabwe; u Desmond Tutu TB Centre, Department of Paediatrics and Child Health, Stellenbosch University, South Africa; v Research Institute for Health Sciences, Chiang Mai University, Chiang Mai, Thailand; w Johns Hopkins University, School of Medicine, Baltimore, MD, USA; x Department of Pediatrics, University of Washington, Seattle, WA, USA

**Keywords:** Pregnancy isoniazid, HEU growth, In utero IPT and growth, IPT and Adverse birth effects, IPT, Isoniazid preventive therapy, LBW, Low birth weight, SGA, Small for gestational age, WAZ, Weight-for-age z-score, WLZ, Weight-for-length z-score, LAZ, Length-for-age z-score, WLWH, Women living with HIV, HEU, HIV-exposed uninfected

## Abstract

**Background:**

Isoniazid preventive therapy (IPT) initiation during pregnancy was associated with increased incidence of adverse pregnancy outcomes in the TB APPRISE trial. Effects of *in utero* IPT exposure on infant growth are unknown.

**Methods:**

This post-hoc analysis used data from the TB APPRISE trial, a multicentre, double-blind, placebo-controlled trial, which randomised women to 28-week IPT starting in pregnancy (pregnancy-IPT) or postpartum week 12 (postpartum-IPT) in eight countries with high tuberculosis prevalence. Participants were enrolled between August 2014 and April 2016. Based on modified intent-to-treat analyses, we analysed only live-born babies who had at least one follow-up after birth and compared time to infant growth faltering between arms to 12 weeks and 48 weeks postpartum in overall and sex-stratified multivariable Cox proportional hazards regression. Factors adjusted in the final models include sex of infant, mother's baseline BMI, age in years, ART regimen, viral load, CD4 count, education, and household food insecurity.

**Results:**

Among 898 HIV-exposed uninfected (HEU) infants, 447 (49.8%) were females. Infants in pregnancy-IPT had a 1.47-fold higher risk of becoming underweight by 12 weeks (aHR 1.47 [95% CI: 1.06, 2.03]) than infants in the postpartum-IPT; increased risk persisted to 48 weeks postpartum (aHR 1.34 [95% CI: 1.01, 1.78]). Maternal IPT timing was not associated with stunting or wasting. In sex-stratified analyses, male infants in the pregnancy-IPT arm experienced an increased risk of low birth weight (LBW) (aRR 2.04 [95% CI: 1.16, 3.68), preterm birth (aRR 1.81 [95% CI: 1.04, 3.21]) and becoming underweight by 12 weeks (aHR 2.02 [95% CI: 1.29, 3.18]) and 48 weeks (aHR 1.82 [95% CI: 1.23, 2.69]). Maternal IPT timing did not influence growth in female infants.

**Interpretation:**

Maternal IPT during pregnancy was associated with an increased risk of LBW, preterm birth, and becoming underweight among HEU infants, particularly male infants. These data add to prior TB APPRISE data, suggesting that IPT during pregnancy impacts infant growth, which could inform management, and warrants further examination of mechanisms.

**Funding:**

The TB APPRISE study Supported by the 10.13039/100000002National Institutes of Health (NIH) (award numbers, UM1AI068632 [IMPAACT LOC], UM1AI068616 [IMPAACT SDMC], and UM1AI106716 [IMPAACT LC]) through the 10.13039/100000060National Institute of Allergy and Infectious Diseases, with cofunding from the 10.13039/100009633Eunice Kennedy Shriver National Institute of Child Health and Human Development (contract number, HHSN275201800001I) and the 10.13039/100000025National Institute of Mental Health.

**Research in context**

**Evidence before this study**

Pregnancy isoniazid preventive therapy (IPT) has not been associated with adverse pregnancy outcomes in observational studies on pregnant women. A rigorously assessed multicenter randomized control trial in women living with HIV on antiretroviral therapy revealed that pregnancy-IPT was associated with an increased incidence of composite adverse pregnancy outcomes (stillbirth, spontaneous abortion, low birth weight (LBW), preterm delivery, and infant congenital anomalies). However, in subsequent secondary analyses of pregnancy cohorts and in a large-scale study of programmatic data in South Africa, antenatal IPT did not appear to be associated with adverse pregnancy outcomes. On the basis of these data, the World Health Organization (WHO) continues to recommend IPT for pregnant women living with HIV, both for their own and their infant's health. In reviewing these studies, we noticed that the long-term and sex-specific effects of pregnancy-IPT on HIV-exposed-uninfected infants were unknown.

**Added value of this study**

This post-hoc analysis of the TB APPRISE trial studied the effects of pregnancy-IPT beyond the previously reported composite pregnancy and birth outcomes to include longer term infant growth outcomes and employed sex-stratified analyses. The data shows that maternal IPT during pregnancy was associated with a significantly increased risk of low birth weight and risk of becoming underweight among HEU infants. Male infants exposed to pregnancy-IPT had a significant risk of low birth weight, preterm birth, and longer-term risk of being underweight that persisted over the first year of life.

**Implications of all the available evidence**

These data add to prior TB APPRISE findings of increased risk of composite adverse pregnancy and birth outcomes associated with IPT during pregnancy, suggesting IPT during pregnancy also impacts the birth size and subsequent infant growth, specifically among male infants. Given that WHO recommended IPT and ART during pregnancy based on data from nonpregnant adults and the absence of harm from observational studies, these data could inform monitoring and management and warrants further examination of potential mechanisms.

## Introduction

Prevention of tuberculosis (TB) among women living with HIV (WLWH) is a high priority in TB endemic areas.^1^ Children born to WLWH who are HIV-exposed but uninfected (HEU) have a higher risk of TB exposure, and TB-related morbidity and mortality compared to HIV-unexposed uninfected (HUU) children.^2^ Thus, the World Health Organization (WHO) recommends TB preventive therapy such as isoniazid preventive therapy (IPT), which reduces the risk of progression from TB infection to TB disease,^3^^,^^4^ for WLWH, both for their own and their infant's health, including during pregnancy.^5^

Observational studies on pregnant women, primarily secondary analyses, did not reveal associations of pregnancy IPT with adverse pregnancy outcomes.^6^^,^^7^ Until recently, safety data regarding IPT in pregnancy rigorously assessed in a trial have been lacking. The TB APPRISE trial evaluated the safety of the immediate (pregnancy-IPT) arm vs. deferred (postpartum-IPT) arm in WLWH on antiretroviral therapy (ART).^8^ In this study, although pregnancy-IPT was as safe as postpartum-IPT with regards to adverse maternal outcomes, pregnancy-IPT was associated with an increased incidence of composite adverse pregnancy outcomes (stillbirth or spontaneous abortion, low birth weight (LBW), preterm delivery, or infant congenital anomalies).^8^

Exposure to HIV and ART in-utero may increase the risk of preterm birth, LBW and low birth length, small for gestational age (SGA), stillbirth,^9^, ^10^, ^11^, ^12^^,^ and growth compromise.^13^ The potential effect of in utero IPT exposure on long-term growth in HEU is not known. Using the TB APPRISE study, we examined the effects of maternal pregnancy-IPT versus postpartum-IPT on growth, and assessed cofactors of growth among HEU in the first year of life. Additionally, we evaluated whether infant sex-modified maternal IPT effect on birth outcomes and infant growth.

## Methods

### Parent trial design and intervention

This post-hoc analysis utilised data from the P1078 TB APPRISE trial – a randomised, double-blind, placebo-controlled, multicentre, non-inferiority study designed to evaluate the effect of pregnancy-IPT vs postpartum-IPT on maternal complications and composite adverse birth outcomes. The trial, as reported in detail previously,^8^ was conducted in eight countries (Botswana, Haiti, India, South Africa, Tanzania, Thailand, Uganda, and Zimbabwe) at 13 different sites with high TB prevalence (>60 cases per 100,000). Participants were randomised to receive a 28-week course of IPT (300 mg daily) either during pregnancy (pregnancy-IPT) or at postpartum week 12 (postpartum-IPT). The pregnancy-IPT arm received isoniazid daily for 28 weeks (initiated between 14- and 34-weeks gestation, immediately after enrolment), then switched to placebo until the 40^th^ week postpartum. The postpartum-IPT arm initiated a placebo immediately after trial entry during pregnancy until the 12th week postpartum and then switched to isoniazid daily until the 40^th^ week postpartum. All participants received vitamin B6 and a prenatal multivitamin from week 0 to week 40 postpartum. The randomisation was stratified by the gestational age at trial entry (≥14 weeks to <24 weeks or ≥24 weeks to ≤34 weeks) and was balanced at each site.

As detailed in the parent paper,^8^ all women provided written informed consent and all local and collaborating institutional review boards approved it. An independent data and safety monitoring board reviewed it biannually. A proposal for these post-hoc analyses was approved by the IMPAACT operations team, and the manuscript was approved for publication by the IMPAACT publication team. The authors attested to the fidelity of the protocol and the accuracy of the analyses. This report conforms with CONSORT reporting guidelines.

### Participants and study period

Pregnant WLWH, 14–34 weeks of gestation, weighing >35 kg, with >750 absolute neutrophil count cells/mm^3^, >7.5 g/dL haemoglobin, and >50,000 platelets count/mm^3^ were eligible. Participants were required to have liver enzymes (aspartate aminotransferase [AST], alanine aminotransferase [ALT], and total bilirubin) at or below 1.25 times the upper limit of the normal range within 30 days prior to study entry. Women with active TB, recent TB exposure, TB treatment for more than 30 days in the previous year, or peripheral neuropathy of grade 1 or higher were excluded. The original study included 956 participants, 477 randomised to pregnancy-IPT and 479 to postpartum-IPT arm. Participants were enrolled between August 2014 and April 2016.

This analysis was restricted to HEU infants born to mothers participating in the RCT. Exclusion criteria for this analysis included lack of infant information (withdrawal from the study before birth or no live birth, or lack of any growth measurement), HIV infection of the infant, and twin births.

### Infant growth characterisation

Mother-infant pairs were followed up to 48 weeks postpartum. Weight and length of infants were measured at birth, 4th, 8th, 12th, 24th, 36th, 44th, and 48th weeks postpartum to the nearest 0.1 kg and 0.1 cm. The scales were calibrated regularly as per the manufacturer's instructions. Shoes and outer layers of clothing were removed before weight measurements were taken. Infants' lengths were measured with horizontal boards. The data collectors were trained and experienced in weight and length measurement. There was a two-week extension period for mothers who did not attend their last visit. Missing values at the scheduled last visit were replaced by measurements within two weeks after the end of the study.

Low birth weight (LBW) was defined as less than 2.5 kg regardless of gestational age. Birth before completion of 37 weeks of pregnancy was regarded as preterm. Small for gestational age (SGA) was defined by weight less than the 10th percentile for gestational age using INTERGROWTH growth standards.^14^

Weight-for-age z-score [WAZ], weight-for-length z-score [WLZ], and length-for-age z-score [LAZ]) were defined using WHO child growth standards.^15^ Growth faltering was less than −2 Z-scores; with underweight defined as WAZ < –2, wasting WLZ < –2, and stunting LAZ < –2.

### Cofactors of growth faltering

Cofactors of growth faltering assessed in the analyses included: Infant sex and maternal characteristics at enrolment, including body mass index (BMI), age, ART regimen, viral non-suppression (viral load ≥40 copies/ml), CD4 count (cells/mm^3^), education, and household food insecurity. Household food insecurity was considered positive if the respondents answered yes to at least one of the following questions: did you experience a lack of resources to get food, have you gone to bed hungry in the last 30 days, and have you passed the entire day and night hungry?

### Statistical analysis

Means and standard deviations (SDs) were used to describe normally distributed continuous variables, medians and interquartile ranges (IQRs) to describe skewed distributions, and frequencies and percentages to describe categorical variables. Baseline maternal and infant characteristics were compared between pregnancy-IPT and postpartum-IPT randomisation groups using two-sided t-tests (Mann–Whitney U tests if assumptions were not met) for continuous variables and Pearson χ^2^ tests (Fisher's exact tests if assumptions were not met) for categorical variables.

In the primary study, randomisation was carried out on pregnant women. The randomisation groups were compared in modified intent-to-treat analyses adjusted for predetermined potential confounding variables.

For the measurement of adverse birth outcomes, the effects of pregnancy-IPT on LBW and preterm birth were examined in the primary trial publication; however, sex-stratified analyses of these outcomes were not conducted. We examined the effects of pregnancy-IPT on birth outcomes (LBW, preterm birth, and SGA) using generalised linear models with a Poisson family and a log link (to estimate relative risks) in overall and sex-stratified analyses. Multivariable generalised linear models were fitted to control potential confounders.

For the measurement of growth faltering during infancy, mothers in the postpartum-IPT arm initiated IPT at 12 weeks after delivery, therefore data were censored at 12-weeks postpartum to examine the effect of pregnancy-IPT compared to no IPT during pregnancy and postpartum. In addition, to compare the longer-term effects of pregnancy-IPT on growth faltering, randomised arms were compared up to 48 weeks after birth. Growth faltering was compared between randomised groups using Cox proportional hazards regression models and generalised estimated equations (GEE).

We used Kaplan–Meier survival analysis to compare, unadjusted, time to the first event of growth faltering (underweight, wasting, or stunting) to 12 weeks postpartum and 48 weeks postpartum in overall and sex-stratified analyses. Univariate and multivariable Cox proportional hazards regression models and models including interaction terms between randomisation arm and infants' sex were fit to compare the risk of experiencing the first episode of growth faltering between the randomised groups and any effect modification by infant sex. For these analyses, time zero was the randomisation date, and no failure (no growth faltering) was assumed prior to birth. Since participants were randomly assigned to either a treatment or a control group during pregnancy, whatever difference occurred between the two arms, such as gestational age at birth or low birth weight, was assumed to be attributable to the intervention. Infants lost to follow-up or who died prior to failure were censored at their last visit date. Growth data from visits following growth faltering were censored. We used multivariate Cox proportional hazards regression models to identify cofactors (maternal BMI, age, ART regimen, viral non-suppression, CD4 count (cells/mm3), education, and household food insecurity and infant sex) of growth faltering. As fewer than 5% of at-risk participants remained in the study after 60 post-randomisation weeks, the values at 60^th^ week and after were censored.

In multivariable models, we didn't adjust birth characteristics because adjusting for low birth weight, preterm birth, and/or SGA would underestimate the effect of the intervention on the outcome as they are in the causal pathway between pregnancy IPT exposure and long-term growth.

Moreover, univariate and multivariable generalised estimated equations (GEE) were fit with a Poisson family and a log link (to estimate relative risk) and exchangeable correlation structure to compare risks of growth faltering (underweight, wasting, and stunting) at any time up until 12 weeks postpartum and up to 48 weeks, as well as testing for interaction by infants' sex and analyses stratified by sex. Infants who experienced growth faltering anytime were not censored in this analysis. The multivariate GEE model was used to identify cofactors of growth faltering in HEU infants.

We also fitted multivariable linear regression to examine the long-term impact of IPT on growth (WAZ, LAZ, WLZ) at 48 weeks of infant age.

We used R version 4.1.0 for analyses.

### Role of the funding source

The funder of the study had no role in study design, data collection, data analysis, data interpretation, or writing of the report. BAR, ASC, and GM had access to the dataset. ASC, SML, GJS, AG, and GM were responsible for the final decision on the submission of this manuscript for publication.

## Results

### Maternal and infant baseline characteristics

**Fig. 1:**
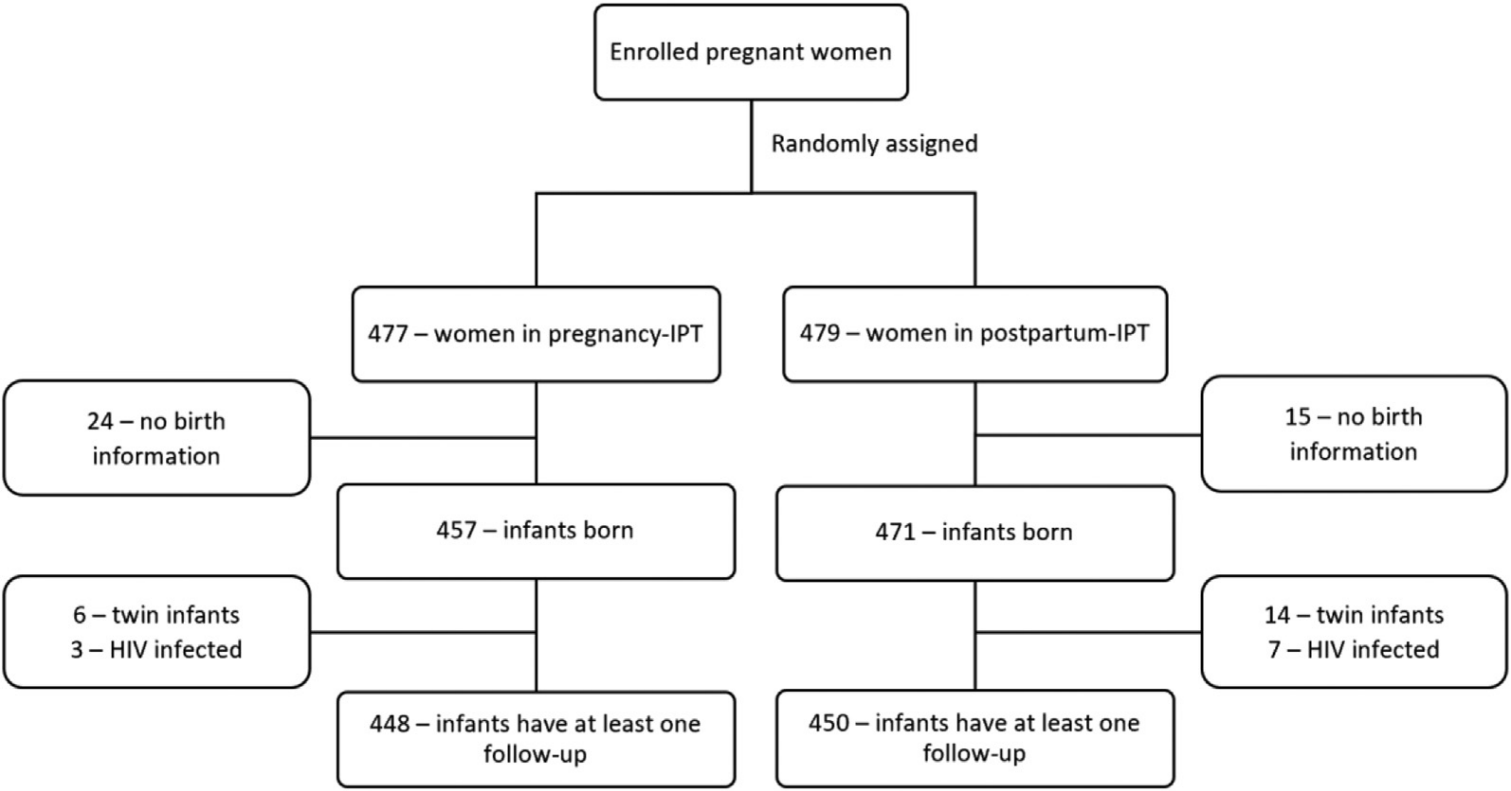
Figure shows enrolment, randomisation, post-hoc exclusion criteria, and analysis. This includes the number of pregnant women randomised to randomisation arms, exclusion criteria, and number of infants included in this post-hoc analysis.

In this study, 898 infants were included: 448 in the pregnancy-IPT and 450 in the postpartum-IPT arm (Fig. 1).

The median age of mothers at enrolment was 29 years (IQR 24–33), and median BMI was 26.2 (IQR 23.3–29.7). All mothers were on ART and the majority received either efavirenz-tenofovir-lamivudine (EFV, TDF, 3TC, 57.3% (480/850)) or efavirenz-tenofovir-emtricitabine (EFV, TDF, FTC, 26.2% (222/850)). Almost half (49.2%) of mothers had a CD4 count above 500 cells/mm3, and 37.6% (316/850) of mothers had a viral load of >40 HIV RNA copies/ml. Almost half (447/898) of the infants were female – 228 in the pregnancy-IPT arm and 219 in the postpartum-IPT arm. Baseline maternal and infant demographic and clinical characteristics were similar between randomised arms (Table 1).

**Table 1:** Baseline maternal and infant demographics and clinical characteristics.

Characteristics	Randomized to start IPT during	
	Pregnancy (n = 448)	Postpartum (n = 450)
Maternal characteristics at baseline		
Median age (IQR) – years	29 (25–33)	29 (24–33)
Education achievement of mothers – no. (%)		
Primary school completed or less	106 (45.7)	126 (54.3)
Secondary school	306 (51.2)	292 (48.8)
Some college education	36 (52.9)	32 (47.1)
Median body mass index (IQR)^a^	26.3 (23.5–30.2)	26.1 (23.1–2.59)
Median CD4 count (IQR)	493.5 (360–678.3)	497 (356.5–665.5)
CD4 count (cells/mm^3^)		
<200	27 (46.6)	31 (53.4)
200-500	201 (50.5)	197 (49.5)
>500	218 (49.5)	222 (50.5)
ART regimen – no. (%)		
Efavirenz–tenofovir–emtricitabine/lamivudine	373 (50.0)	373 (50.0)
Efavirenz–zidovudine–lamivudine	3 (27.3)	8 (72.7)
Nevirapine–zidovudine or tenofovir–emtricitabine or lamivudine	58 (49.2)	60 (50.8)
Lopinavir or atazanavir–ritonavir with tenofovir or Zidovudine–emtricitable or lamivudine	12 (63.2)	7 (36.8)
Efavirenz only	1	0
Median HIV-1 RNA copies/ml (IQR)	39 (39–107)	39 (39–138)
Viral load (≥40 HIV copies/ml) – no. (%)	165 (36.9)	173 (38.5)
Cotrimoxazole use – no. (%)	198 (44.3)	189 (42.1)
Positive IGRA status – no./total no. (%)	127/442 (28.7)	134/445 (30.1)
Mean gestation age at enrollment ± sd	26.1 ± 5.3	25.8 + 5.3
Infant characteristics at birth		
Female infants – no. (%)	228 (49.1)	219 (51.3)
Mean birthweight in kilogram (SD)	3.0 (0.6)	3.0 (0.6)
Low birthweight – no./total no. (%)^b^	59/425 (13.9)	40/433 (9.2)
Preterm birth – no. (%)^c^	54 (12.1)	42 (9.3)
Small for gestational age – no./total no. (%)^d^	79/425 (18.6)	86/433 (19.7)

a Body mass index is the weight in kilograms divided by the square of height in meters.

b Low birth weight is an infant born weighing 5.5 pounds (2.5 kg) or less.

c Preterm is a baby born before the 37th week of gestation.

d Small for gestation age is defined as weight less than 10th percentile for gestational age using intergrowth growth standards.

### Maternal and infant TB status throughout the study period

As reported in the primary analysis, six mothers (0.4%) and one infant (0.1%) developed TB, and 241 mothers (32.4%) and 41 (5.8%) infants tested positive on a QuantiFERON-TB Gold In-Tube (QGIT) test. There was no significant difference in TB disease or infection incidence in mothers or infants between arms.

### Effect of IPT on WAZ, LAZ, and WLZ at 48 weeks of infants age

There was no unadjusted mean difference in WAZ (B 0.04 [95% CI: −0.15, 0.23], p = 0.67), LAZ (B −0.04 [95% CI: −0.34, 0.27], p = 0.81), and WLZ (B 0.13 [95% CI: −0.09, 0.35], p = 0.24) at 48 weeks of infants age between pregnancy-IPT and postpartum-IPT. Similarly, there was no adjusted mean difference in WAZ (B 0.00 [95% CI: −0.18, 0.18], p = 0.99), LAZ (B −0.10 [95% CI: −0.41, 0.21], p = 0.52), and WLZ (B 0.11 [95% CI: −0.10, 0.33], p = 0.30) at 48 weeks of infants age between pregnancy-IPT and postpartum-IPT.

### Effect of pregnancy-IPT on adverse birth outcomes – underweight, preterm, and SGA

Overall, 10.7% of infants were premature, 11.5% were LBW, and 19.2% were SGA at birth. Adjusted for relevant cofactors, infants in the pregnancy-IPT arm had a 1.60-fold higher risk of LBW (aRR 1.60 [95% CI: 1.07, 2.41]) than infants in the postpartum-IPT arm (Table 2). There was no significant difference in risk of being preterm (aRR 1.31 [95% CI: 0.87, 1.97]) or SGA (aRR 0.97 [95% CI: 0.71, 1.32]) between randomisation arms.

**Table 2:** Risks of low birth weight, preterm, and small for gestational age overall and stratified by infant sex.

Analysis	Model	Isoniazid started during	Low birthweight^c^			Preterm^d^			Small for gestational age^e^		
			cRR^a^ (95% CI)	aRR^b^ (95% CI)	P-value	cRR^a^ (95% CI)	aRR^b^ (95% CI)	P-value	cRR^a^ (95% CI)	aRR^b^ (95% CI)	P-value
**Birth**	Overall	Pregnancy	1.50 (1.01, 2.26)	1.60 (1.07, 2.41)	0.022	1.29 (0.86, 1.94)	1.31 (0.87, 1.97)	0.20	0.94 (0.69, 1.27)	0.96 (0.71, 1.31)	0.82
		Postpartum	1	1		1	1		1	1	
	Male infants	Pregnancy	1.87 (1.08, 3.35)	2.04 (1.16, 3.68)	0.015	1.79 (1.04, 3.15)	1.81 (1.04, 3.21)	0.038	1.12 (0.74, 1.69)	1.21 (0.79, 1.84)	0.37
		Postpartum	1	1		1	1		1	1	
	Female infants	Pregnancy	1.18 (0.66, 2.13)	1.25 (0.69, 2.27)	0.47	0.87 (0.47, 1.60)	0.94 (0.50, 1.75)	0.85	0.75 (0.47, 1.19)	0.70 (0.43, 1.12)	0.15
		Postpartum	1	1		1	1		1	1	

a cRR – crude relative risk.

b aRR – relative risk-adjusted for maternal body mass index (weight in kilograms divided by the square of height in meters), age in years, ART regimen, viral suppression, CD4 count, education, and household food insecurity.

c Low birth weight is an infant born weighing 5.5 pounds (2.5 kg) or less.

d Preterm is a baby born before the 37th week of gestation.

e Small for gestation age is defined as weight less than 10th percentile for gestational age using intergrowth growth standards.

In sex-stratified analyses adjusted for all cofactors, male infants in the pregnancy-IPT arm had a 2.04-fold higher risk of LBW (aRR 2.04 [95% CI: 1.16, 3.68) and 1.81-fold increased risk of preterm birth (aRR 1.81 [95% CI: 1.04, 3.21]) than those in the postpartum-IPT arm. Pregnancy-IPT was not associated with LBW or preterm delivery among female infants or SGA among male and female infants (Table 2).

### Risk of experiencing growth faltering during infancy

The overall risk of being underweight during the 48-week follow-up period was 22.8 per 100 person-years (95% CI: 19.4, 26.0), risk of stunting was 40.1 per 100 person-years (95% CI: 35.9, 44.1), and risk of wasting was 32.8 per 100 person-years (95% CI: 28.9, 36.5).

**Fig. 2:**
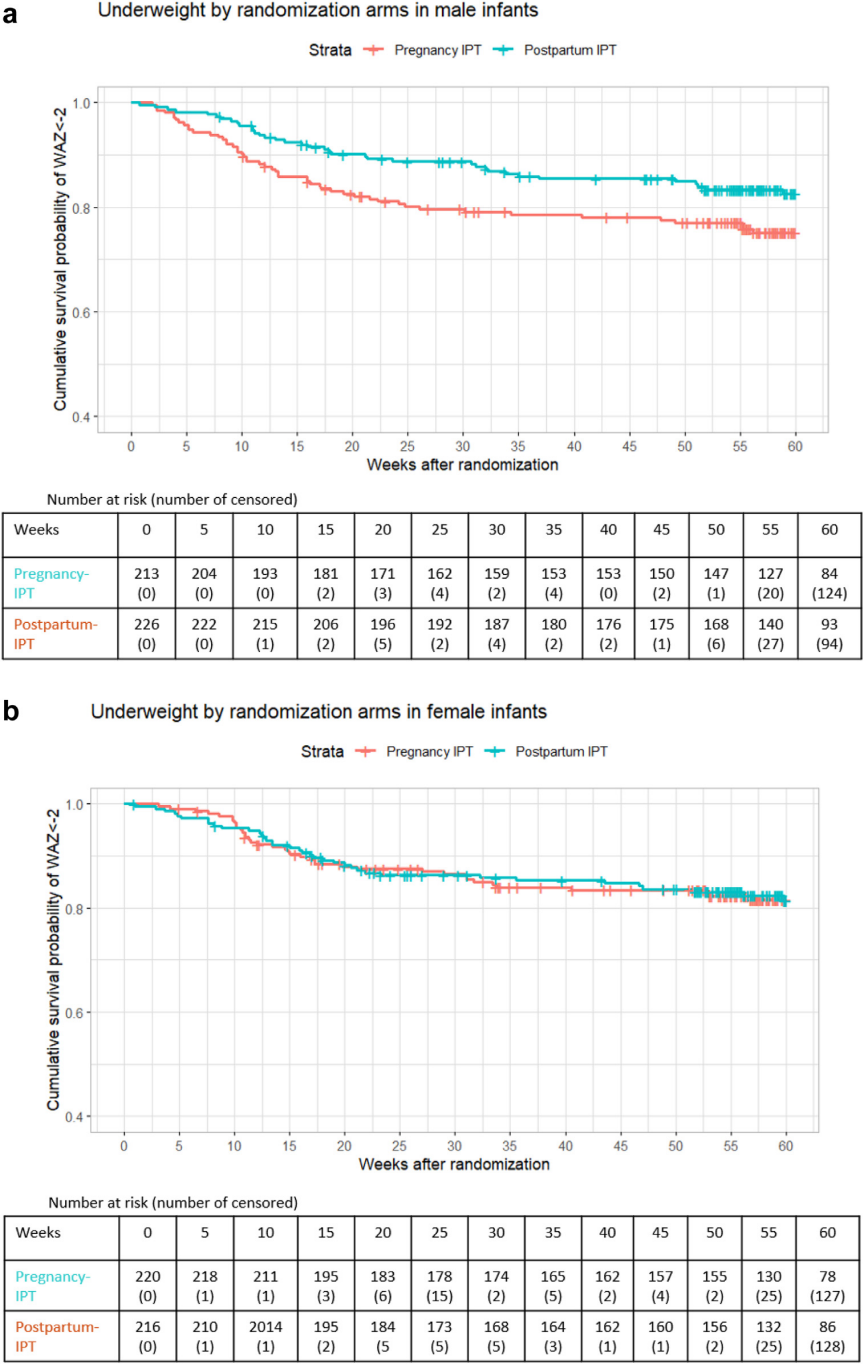

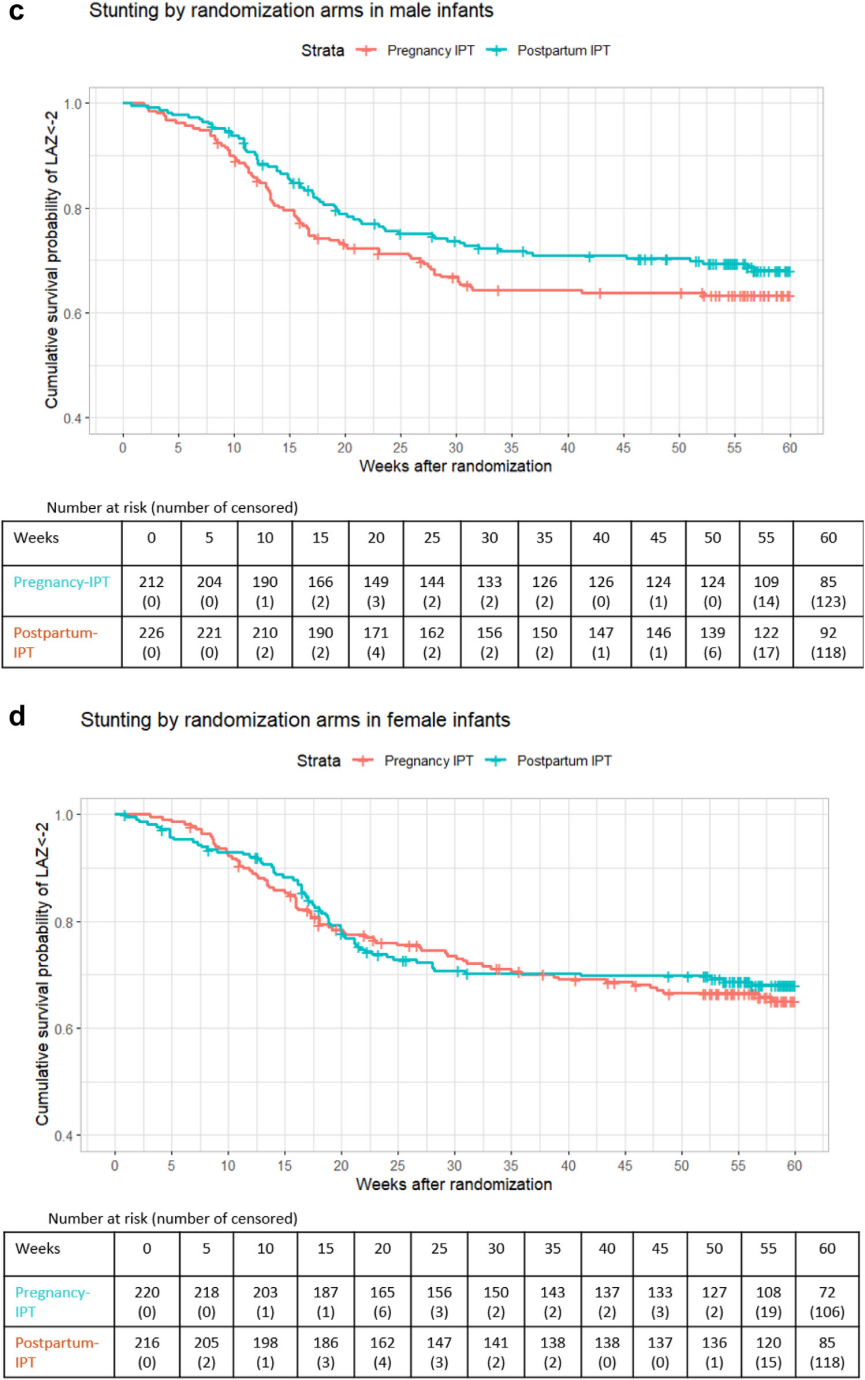

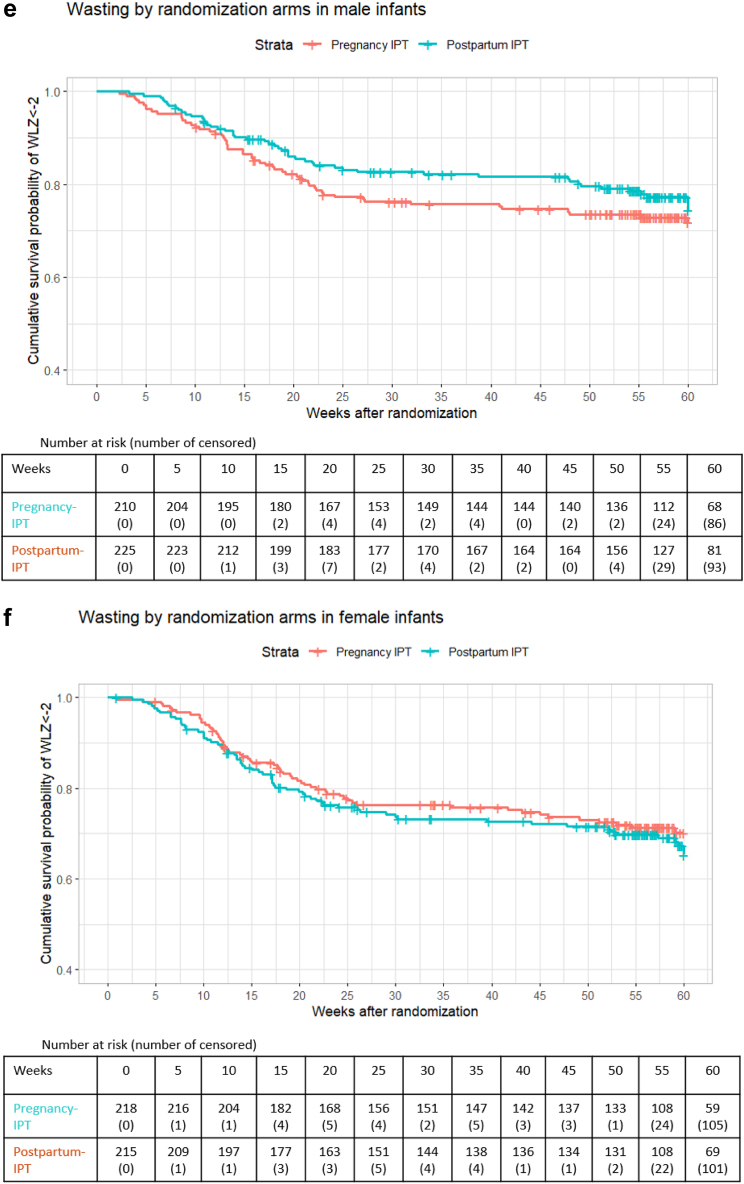
Figure includes the Kaplan–Meier curves that show sex-stratified cumulative survival probability of underweight (defined as weight-for-age (WAZ)<-2), wasting (defined as weight-for-length (WLZ)<-2), and stunting (defined as length-for-age (LAZ)<-2) by randomised arms. For these Kaplan–Meier, time 0 was the randomisation date during pregnancy. (a) includes the Kaplan–Meier curves that show cumulative survival probability of underweight (defined as weight-for-age (WAZ)<-2) by randomised arms in male infants. For these Kaplan–Meier, time 0 was the randomisation date during pregnancy. (b) includes the Kaplan–Meier curves that show cumulative survival probability of underweight (defined as weight-for-age (WAZ)<-2) by randomised arms in female infants. For these Kaplan–Meier, time 0 was the randomisation date during pregnancy. (c) includes the Kaplan–Meier curves that show cumulative survival probability of stunting (defined as length-for-age (LAZ)<-2) by randomised arms in male infants. For these Kaplan–Meier, time 0 was the randomisation date during pregnancy. (d) includes the Kaplan–Meier curves that show cumulative survival probability of stunting (defined as length-for-age (LAZ)<-2) by randomised arms in female infants. For these Kaplan–Meier, time 0 was the randomisation date during pregnancy. (e) includes the Kaplan–Meier curves that show cumulative survival probability of wasting (defined as weight-for-length (WLZ)<-2) by randomised arms in male infants. For these Kaplan–Meier, time 0 was the randomisation date during pregnancy. (f) includes the Kaplan–Meier curves that show cumulative survival probability of wasting (defined as weight-for-length (WLZ)<-2) by randomised arms in female infants. For these Kaplan–Meier, time 0 was the randomisation date during pregnancy.

In univariate analysis, male infants in the pregnancy-IPT had a significantly higher cumulative probability of being underweight (30.5 per 100 person-years [95% CI: 22.02, 38.1] vs 19.7 per 100 person-years [95% CI: 13.5, 25.5]), p = 0.041) than male infants in the postpartum-IPT arm (Fig. 2a). Fig. 2a–f illustrate Kaplan–Meier curves stratified by sex.

In multivariable Cox regression models, pregnancy-IPT was associated with infant underweight in analyses to 12 weeks and 48 weeks postpartum. Infants in the pregnancy-IPT arm experienced a 1.47-fold higher risk of becoming underweight in the first 12 weeks (aHR 1.47 [95% CI: 1.06, 2.03]) and a 1.34-fold higher risk of becoming underweight in the first 48 weeks (aHR 1.34 [95% CI: 1.01, 1.78]) than infants in the postpartum-IPT arm. Maternal IPT timing was not associated with stunting (aHR by 12 weeks 1.12 [95% CI: 0.91, 1.39] and aHR by 48 weeks 1.08 [95% CI: 0.89, 1.30]) or wasting (aHR by 12 weeks 1.09 [95% CI: 0.81, 1.45] and aHR by 48 weeks 1.02 [95% CI: 0.79, 1.32]) (Table 3).

**Table 3:** Risks of growth faltering overall and stratified by infant sex in analyses to 12 and 48 weeks postpartum.

			Underweight^a^			Stunting^b^			Wasting^c^		
			cHR^d^ (95% CI)	aHR^e^ (95% CI)	P-value	cHR^d^ (95% CI)	aHR^e^ (95% CI)	P-value	aHR^d^ (95% CI)	aHR^e^ (95% CI)	P-value
**12-week postpartum**	Overall	Pregnancy	1.37 (0.99, 1.89)	1.47 (1.06, 2.03)	0.021	1.10 (0.89, 1.36)	1.12 (0.91, 1.39)	0.28	1.11 (0.83, 1.47)	1.09 (0.81, 1.45)	0.57
		Postpartum	1	1		1	1		1	1	
	Male infants	Pregnancy	1.83 (1.18, 2.84)	2.02 (1.29, 3.18)	0.0022	1.21 (0.91, 1.62)	1.22 (0.91, 1.64)	0.19	1.43 (0.94, 2.19)	1.61 (1.04, 2.49)	0.031
		Postpartum				1	1		1	1	
	Female infants	Pregnancy	0.97 (0.60, 1.56)	0.97 (0.59, 1.58)	0.89	0.98 (0.72, 1.34)	0.98 (0.71, 1.35)	0.90	0.88 (0.60, 1.29)	0.76 (0.51, 1.12)	0.17
		Postpartum	1	1		1	1		1	1	
**48 weeks postpartum**	Overall	Pregnancy	1.26 (0.96, 1.67)	1.34 (1.01, 1.78)	0.042	1.05 (0.86, 1.26)	1.08 (0.89, 1.30)	0.46	1.03 (0.80, 1.33)	1.02 (0.79, 1.32)	0.87
		Postpartum	1	1		1	1		1	1	
	Male infants	Pregnancy	1.66 (1.13, 2.44)	1.82 (1.23, 2.69)	0.0027	1.11 (0.85, 1.45)	1.13 (0.86, 1.48)	0.38	1.23 (0.84, 1.79)	1.40 (0.95, 2.06)	0.091
		Postpartum	1	1		1	1		1	1	
	Female infants	Pregnancy	0.92 (0.61, 1.40)	0.90 (0.59, 1.38)	0.63	0.99 (0.75, 1.30)	1.00 (0.75, 1.33)	0.99	0.88 (0.62, 1.25)	0.79 (0.55, 1.13)	0.20
		Postpartum	1	1		1	1		1	1	

a Underweight – defined as weight-for-age (WAZ)<-2).

b Wasting – defined as weight-for-length (WLZ)<-2.

c Stunting – defined as length-for-age (LAZ)<-2.

d cHR – crude hazard ratio.

e HRa – hazard ratio adjusted for maternal body mass index (weight in kilograms divided by the square of height in meters), age in years, ART regimen, viral suppression, CD4 count, education, and household food insecurity.

Infant sex significantly modified the effect of pregnancy-IPT on underweight in analyses to 12 weeks (p-value = 0.037) and 48 weeks (p-value = 0.022). Male infants in the pregnancy-IPT arm experienced a 2.02-fold increased risk of becoming underweight in the first 12 weeks (aHR 2.02 [95% CI: 1.29, 3.18) and a 1.82-fold increased risk of becoming underweight in the first 48 weeks (aHR 1.82 [95% CI: 1.23, 2.69]) than male infants in the postpartum-IPT arm (Table 3).

There was also a statistically significant differential effect of pregnancy-IPT on wasting in male and female infants by 12 weeks (p-value = 0.021), but not by 48 weeks (p-value = 0.057). Male infants in the pregnancy-IPT arm experienced a 1.61-fold higher risk of becoming wasted in the first 12 weeks (aHR 1.61 [95% CI: 1.04, 2.49), and a 1.43-fold higher risk (nonsignificant) of becoming wasted in the first 48 weeks (aHR 1.40 [95% CI: 0.95, 2.06) than male infants in the postpartum-IPT arm (Table 3).

Among female infants, pregnancy-IPT was not associated with growth faltering – underweight (aHR at 12 weeks 0.97 [95% CI: 0.59, 1.58] and aHR at 48 weeks 0.90 (95% CI: 0.59, 1.38]), stunting (aHR at 12 weeks 0.98 [95% CI: 0.71, 1.35] and aHR at 48 weeks 1.00 [0.75, 1.33), and wasting (aHR at 12 weeks 0.76 [95% CI: 0.51, 1.12] and aHR at 48 weeks 0.79 [95% CI: 0.55, 1.13]).

We also fitted GEE multivariable models to estimate the repeated prevalence of growth faltering during infancy which yielded similar results to Cox regression analyses (Supplemental Table S1).

### Cofactors of growth faltering

For every 1 kg/m^2^ increase in maternal BMI, infant risk of being underweight decreased by 7% (aHR 0.93 [95% CI: 0.90, 0.96]), and wasting risk decreased by 8% (aHR 0.92 [95% CI: 0.89, 0.94]) (Table 4).

**Table 4:** Cofactors of risk of growth faltering in the overall cohort of HEU infants in analysis to 48 weeks postpartum.

Variables	Underweight^a^			Stunting^b^			Wasting^c^		
	cHR^d^ (95% CI)	aHR^e^ (95% CI)	P-value	cHR^d^ (95% CI)	aHR^e^ (95% CI)	P-value	aHR^d^ (95% CI)	aHR^e^ (95% CI)	P-value
IPT started during pregnancy	1.26 (0.96, 1.67)	1.34 (1.01, 1.78)	0.042	1.05 (0.86, 1.26)	1.08 (0.89, 1.30)	0.46	1.06 (0.82, 1.38)	1.05 (0.81, 1.37)	0.72
Male infant	1.23 (0.93, 1.63)	1.27 (0.96, 1.69)	0.095	1.14 (0.94, 1.37)	1.14 (0.94, 1.38)	0.20	0.80 (0.61, 1.03)	0.82 (0.63, 1.07)	0.14
Mother's BMI^f^	0.93 (0.90, 0.96)	0.93 (0.90, 0.96)	<0.0001	0.98 (0.96, 1.00)	0.98 (0.96, 1.00)	0.075	0.93 (0.90, 0.96)	0.92 (0.89, 0.95)	<0.0001
Age of a mother in years	1.00 (0.97, 1.02)	1.00 (0.97, 1.03)	0.99	0.98 (0.96, 1.00)	0.98 (0.96, 1.00)	0.035	1.03 (1.01, 1.05)	1.04 (1.01, 1.06)	0.0036
Art regimen			0.20			0.093			0.20
3TC/FTC,TDF,EFV	1	1		1	1		1	1	
3TC, ZDV, EFV	0.82 (0.20, 3.29)	0.89 (0.22, 3.65)		2.03 (1.01, 4.10)	1.91 (0.93, 3.91)		–	–	
3TC/FTC, LVP/ATV, TDF/ZDV	0.96 (0.35, 2.58)	0.82 (0.30, 2.24)		1.36 (0.75, 2.48)	1.32 (0.72, 2.43)		1.14 (0.47, 2.77)	1.10 (0.45, 2.70)	
3TC/FTC, ZDV/TDF, NVP	1.36 (0.93, 1.99)	1.59 (1.06, 2.37)		1.20 (0.91, 1.57)	1.34 (1.00, 1.79)		1.09 (0.75, 1.58)	1.13 (0.77, 1.67)	
Viral load (≥40 HIV copies/ml)	1.07 (0.81, 1.43)	1.11 (0.80, 1.51)	0.55	1.11 (0.92, 1.36)	1.13 (0.91, 1.40)	0.26	0.90 (0.68, 1.17)	0.83 (0.62, 1.12)	0.23
CD4 count (cells/mm^3^)			0.30			0.90			0.60
<200	1	1		1	1		1	1	
200–500	0.71 (0.42, 1.22)	0.65 (0.37, 1.12)		0.95 (0.64, 1.40)	0.91 (0.61, 1.36)		0.92 (0.55, 1.54)	0.87 (0.52, 1.47)	0.61
>500	0.80 (0.47, 1.36)	0.75 (0.43, 1.32)		0.98 (0.66, 1.44)	0.94 (0.62, 1.41)		0.81 (0.49, 1.36)	0.78 (0.45, 1.34)	0.37
Mother's education			0.40			0.080			0.057
Primary school completed or less	1	1		1	1		1	1	
Secondary school	0.91 (0.67, 1.25)	0.88 (0.63, 1.22)		0.79 (0.64, 0.98)	0.82 (0.65, 1.02)		1.49 (1.07, 2.07)	1.45 (1.04, 2.03)	
Some college education	0.66 (0.34, 1.26)	0.65 (0.33, 1.26)		0.70 (0.47, 1.05)	0.66 (0.43, 1.01)		1.26 (0.71, 2.23)	1.30 (0.73, 2.34)	
Food insecure household	0.94 (0.61, 1.45)	0.87 (0.56, 1.36)	0.55	1.10 (0.83, 1.46)	1.04 (0.78, 1.39)	0.78	0.91 (0.61, 1.37)	0.91 (0.60, 1.38)	0.65

a Underweight – defined as weight-for-age (WAZ)<-2).

b Wasting – defined as weight-for-length (WLZ)<-2.

c Stunting – defined as length-for-age (LAZ)<-2.

d cHR – crude hazard rate.

e aHR – hazard ratio adjusted for maternal body mass index (weight in kilograms divided by the square of height in meters), age in years, ART regimen, viral suppression, CD4 count, education, and household food insecurity.

f Body mass index is the weight in kilograms divided by the square of height in meters.

For every additional year of maternal age, the risk of wasting in infants increased by 4% (aHR 1.04 [95% CI: 1.01, 1.06]), while the risk of stunting decreased by 2% (aHR 0.98 [95% CI: 0.96, 1.00]). Infants born to mothers who used NVP-regimens experienced 1.6-fold increased risk of being underweight (aHR 1.62 [95% CI: 1.09, 2.41]) and 1.34 increased risk of stunting (aHR 1.34 [95% CI: 1.00, 1.79]) compared to EFV, TDF, 3TC/FTC regimens. Infants born from mothers who have secondary education have a 21% lower risk of stunting (aHR 0.79 [95% CI: 0.64, 0.98]) but a 45% higher risk of wasting (aHR 1.45 [1.04, 2.03]) (Table 4).

Multivariable GEE models used to assess cofactors of growth faltering yielded similar results as Cox proportional hazard regression models above (Supplemental Table S2).

## Discussion

In this post-hoc analysis of a multi-site RCT evaluating maternal IPT in pregnancy versus postpartum, timing of maternal IPT influenced growth outcomes among HEU infants with a significantly higher risk of underweight among infants born to mothers in the pregnancy-IPT arm. There was an effect modification of associations of IPT with growth by infant sex, with significantly increased underweight and wasting in males born to mothers in the pregnancy-IPT versus postpartum-IPT arms. Our findings suggest that the timing of maternal IPT may influence birth size as well as postnatal growth in infants and provide valuable data for policymakers and clinicians considering the optimal timing of IPT in pregnant WLWH.

Our data suggest growth-altering effects of in utero IPT exposure. Infants born to mothers randomised to pregnancy-IPT had a 1.60-fold higher risk of LBW and of becoming underweight during the first year of life than infants born to mothers randomised to postpartum-IPT. During pregnancy, IPT causes embryocidal effects on rats and rabbits, delays neurodevelopment in zebrafish, and affects postnatal growth, development, and cognitive ability in rats.^16^, ^17^, ^18^ In vitro studies have demonstrated cytotoxic effects of IPT that disturb the cell cycle in mammalian cells.^19^ Antenatal IPT may induce poor appetite, nausea, emesis, and hepatic changes in the mother that could, in turn, affect infant growth.^3^^,^^20^ In addition, since isoniazid crosses placenta barrier,^16^ direct drug effects on the Foetus could influence growth.

We found that associations between pregnancy-IPT and growth were modified by infant sex. Male infants in the pregnancy-IPT arm had a significantly higher risk of preterm birth, LBW, and longer-term growth faltering than male infants in the postpartum-IPT arm. In contrast, birth outcomes and growth in female infants were not affected by pregnancy-IPT. In utero growth trajectories differ by sex; male foetuses typically grow faster and may not alter their growth trajectory when facing adverse challenges.^21^, ^22^, ^23^, ^24^, ^25^ Due to the continued growth without adaptations early in pregnancy, male Foetus exposed to adverse intrauterine exposure (maternal/ecological/environmental challenges) may have adverse outcomes later in pregnancy,^22^^,^^23^ including growth restriction,^26^^,^^27^ than female infants. Placental gene transcription differences,^23^^,^^28^ prenatal testosterone exposure,^29^ differences in cellular genes (XX-specific versus XY-specific),^22^^,^^23^^,^^28^^,^^29^ and maternal glucocorticoids^23^ influence sex-differential adaptation response to intrauterine exposure. These factors may contribute to sex-differential growth effects of in utero IPT. Our findings also suggest that in utero IPT continues to affect male growth after birth. Similarly, mixed-twin studies show male susceptibility: male twins are more likely to experience congenital anomalies and a higher risk of infant mortality and neonatal morbidity than their female twins.^30^

Pregnancy-IPT was not associated with overall or sex-stratified SGA. The fact that male infants in pregnancy-IPT had a 1.81 higher risk of preterm birth while there was no difference in SGA risk than male infants in postpartum-IPT suggests that the mechanism for LBW in male infants may be through preterm birth.

In addition to our primary goal of defining the impact of pregnancy IPT exposure on infant growth, we assessed other cofactors of growth in HEU infants. We found expected associations between maternal BMI and being underweight. Nutritional status during pregnancy affects the Foetus's nutrition, potentially altering its growth. We also found that stunting risk decreased by 2% for every additional year of maternal age. This was consistent with a study using data from 18 countries' Demographic Health Surveys, which found that infants of young mothers had lower heights than infants born to older mothers.^31^ Infants of young mothers may be more prone to intrauterine growth restriction.^21^ The maternal use of nevirapine regimens was associated with growth faltering; potential mechanisms for this association are unclear.

This study has multiple strengths, including randomised allocation of IPT timing, excellent retention, and sufficient sample size to investigate growth outcomes. The study also has limitations. This post-hoc analysis was designed after the primary RCT was completed. After 12 weeks of age, infants in the deferred arm received postpartum IPT, so there is not a no-IPT comparator after this time point. However, in analyses that excluded the time period with exposure to postpartum IPT, growth effects of pregnancy-IPT remained similar. Because of the lack of information about infants' growth during pregnancy, we assumed no failure (no growth faltering) prior to birth, and birth was assumed as time-one, which could be a limitation. Infants with compromised growth before delivery would have been censored before birth if we had this prior birth information. As a result, it is possible that some infants might have switched categories before birth as a result - from compromised growth to normal growth and vice versa. However, we strongly believe that the design of our study has addressed/minimised the impact of this on the results of our study. Given that randomisation was applied, any difference between the two arms is attributed to IPT exposure during pregnancy. Similarly, the double-blind placebo-controlled randomised controlled trial design has addressed/minimised potential bias and measurement errors. Randomization has addressed selection bias. To reduce measurement error, data collectors were trained in how to measure height and weight in infants. As it was a double-blinded and placebo-controlled design, even if measurement error occurred, there would have been non-differential misclassification, which would attenuate the effect of the exposure on the outcome. Therefore, the design allows us to ensure that our findings are not biased or influenced by measurement errors (if anything, they are attenuated). And, randomisation was balanced by study site to account for heterogeneity.

Breastfeeding is crucial for infant development and growth. We did not include breastfeeding-related variables in our models for the following reasons: 1) to avoid adjusting a potential mediator: Preterm birth and low birth weight are hypothesised pathways to long-term growth effects, and both affect breastfeeding negatively, which make breastfeeding part of the potential causal pathway. As a result, adjusting breastfeeding variables would attenuate intervention effect. 2) Differential censorship: Randomisation date during pregnancy was time zero, and breastfeeding is a time-varying variable that can only be collected after birth. The association between breastfeeding and growth would exclude infants with restricted growth at birth due to censoring in the survival analysis design. 3) Left truncation bias: If adverse birth outcomes affect breastfeeding practice and a large number of infants with adverse birth outcomes are censored at birth (time 1), including breastfeeding in our models to assess its association with growth over each follow-up period would introduce bias. We would be able to fit breastfeeding variables only to infants who survived time one (infants without adverse birth outcomes). For these reasons adding breastfeeding variables to our models would be problematic." As a result of these reasons, and the fact that only a few infants had concurrent infections at each follow-up (on average less than 4 infants had pneumonia), we did not include infection status in the analysis.

Other important factors that affect the growth of infants during pregnancy are the drinking and smoking experiences of mothers. Due to the very small number of mothers smoking at enrolment (<2%), we did not include maternal smoking experience as a cofactor in the model. Mothers' drinking experience at enrolment or after was not collected. Nonetheless, the results of the study regarding the effects of IPT on growth would not have been affected by these variables since randomisation would distribute them evenly between the randomisation arms.

In conclusion, in this post-hoc analysis, maternal IPT during pregnancy was associated with a significantly increased risk of LBW and risk of becoming underweight among HEU infants. Male infants exposed to pregnancy-IPT had a significant risk of LBW, preterm birth, and longer-term risk of being underweight that persisted over the first year of life. These data add to prior TB APPRISE findings of increased risk of composite adverse birth outcomes associated with IPT during pregnancy, suggesting IPT during pregnancy also impacts birth size and infant growth specifically among male infants. These data could inform monitoring and management and warrants further examination of potential mechanisms.

## Contributors

ACH participated in the investigation of the parent study and reviewed and edited this manuscript.

AG designed and analysed the parent study. Reviewed and edited this manuscript.

ASC∗ participated in all activities of this post-hoc analysis: data management, analysis, writing and editing the manuscript.

AW participated in the investigation of the parent study and reviewed and edited this manuscript.

BAR∗ participated in all activities of this post-hoc analysis: data management, analysis, writing and editing the manuscript.

BTM participated in the investigation of the parent study and reviewed and edited this manuscript.

DAE participated in all activities of this post-hoc analysis: data management, analysis, writing and editing the manuscript.

DW participated in the investigation and validation of the parent study and reviewed and edited this manuscript.

EJ participated in the investigation and validation of the parent study and reviewed and edited this manuscript.

GM participated in the investigation of the parent study and reviewed and edited this manuscript.

GM∗ designed/analysed parent study, data curation, and methodology and reviewed & edited the manuscript.

GT participated in the investigation of the parent study and reviewed and edited this manuscript.

GJS participated in all activities of this post-hoc analysis: data management analysis, writing and editing the manuscript.

HC participated in the investigation of the parent study and reviewed and edited this manuscript.

LA participated in the investigation of the parent study and reviewed and edited this manuscript.

LSC participated in the investigation of the parent study and reviewed and edited this manuscript

MSR participated in the investigation and validation of the parent study and reviewed and edited this manuscript.

NC participated in the investigation of the parent study and reviewed and edited this manuscript.

PJP participated in the investigation of the parent study and reviewed and edited this manuscript.

SB participated in protocol development and study implementation, and operational support.

SML participated in all activities of this post-hoc analysis: data management, analysis, writing and editing manuscript.

TN participated in the investigation and validation of the parent study and reviewed and edited this manuscript.

TM participated in the investigation and validation of the parent study and reviewed and edited this manuscript.

∗ASC, BAR, and GM have accessed and verified the underlying data.

## Data sharing statement

Due to ethical restrictions in the informed consent documents and in the approved human subjects protection plan of the International Maternal Pediatric Adolescent AIDS Clinical Trials (IMPAACT) Network, the study's data cannot be made publicly available; public availability could compromise participant privacy. However, data are available to all interested researchers upon request to the IMPAACT Statistical and Data Management Center's data access committee (email address: sdac.data@fstrf.org) with the agreement of the IMPAACT Network.

## Declaration of interests

AW declares grants from GSK, Merck, and Janssen; payment for expert testimony from GSK and Merck; and participation on a data safety monitoring board for GSK, Merck, and Seqirus. All other authors declare no competing interests.

## Appendix A Supplementary data

Supplementary Tables S1 and S2

IMPAACT P1078 TB APPRISE Study Team
