# Supplementary material for: Effect of pregnancy versus postpartum maternal isoniazid preventive therapy on infant growth in HIV-exposed uninfected infants: a post-hoc analysis of the TB APPRISE trial
Source: eClinicalMedicine. 2023 Mar 17;58:101912. doi: 10.1016/j.eclinm.2023.101912 (PMC10031034; doi:10.1016/j.eclinm.2023.101912)
Supplement: Supplementary Tables S1 and S2 [file mmc1.pdf]

Supplemental

Supplemental Table 1: Risk of underweight, stunting, and wasting overall and stratified by infant sex in analysis to 12 and 48 weeks postpartum using GEE models with recurrent outcomes

Analysis	Model	Isoniazid started during	Underweight[s]			Stunting[¶]			Wasting[β]		
			cRR[¥] (95% CI)	aRR[¤] (95% CI)	P-value	cRR[¥] (95% CI)	aRR[¤] (95% CI)	P-value	cRR[¥] (95% CI)	aRR[¤] (95% CI)	P-value
**12-week postpartum**	Overall	Pregnancy	**1·38 (1·00, 1·91)**	**1·47 (1·07, 2·02)**	**0·017**	1·14 (0·95, 1·38)	1·18 (0·98, 1·42)	0·08	1·08 (0·82, 1·44)	1·06 (0·81, 1·39)	0·65
		Postpartum	1	1		1	1		1	1	
	Male infants	Pregnancy	**1·68 (1·11, 2·56)**	**1·78 (1·18, 2·69)**	**0·0060**	1·27 (1·00, 1·63)	1·27 (0·99, 1·63)	0·058	1·42 (0·95, 2·13)	**1·63 (1·10, 2·42)**	**0·015**
		Postpartum	1	1		1	1		1	1	
	Female infants	Pregnancy	1·04 (0·63, 1·72)	1·09 (0·66, 1·81)	0·73	1·01 (0·76, 1·33)	1·02 (0·76, 1·36)	0·92	0·91 (0·62, 1·33)	**0·82 (0·56, 1·20)**	**0·31**
		Postpartum	1	1		1	1		1	1	
**48 weeks postpartum**	Overall	Pregnancy	1·28 (0·97, 1·70)	**1·36 (1·04, 1·78)**	**0·027**	1·12 (0·94, 1·34)	1·16 (0·97, 1·39)	0·10	0·98 (0·75, 1·29)	0·98 (0·75, 1·27)	0·86
		Postpartum	1	1		1	1		1	1	
	Male infants	Pregnancy	**1·59 (1·09, 2·32)**	**1·69 (1·19, 2·41)**	**0·0040**	1·23 (0·97, 1·56)	1·25 (0·98, 1·59)	0·07	1· 34 (0·89, 2·02)	1·48 (1·00, 2·21)	0·052
		Postpartum	1	1		1	1		1	1	
	Female infants	Pregnancy	0·98 (0·64, 1·50)	1·01 (0·67, 1·52)	0·97	1·00 (0·76, 1·30)	1·01 (0·77, 1·32)	0·95	0·75 (0·52, 1·08)	**0·68 (0·47, 0·99)**	**0·042**
		Postpartum	1	1		1	1		1	1	

[s]Underweight – defined as weight-for-age (WAZ)<-2). [¶]Wasting – defined as weight-for-length (WLZ)<-2. [β]Stunting – defined as length-for-age (LAZ)<-2. [¥]cRR – crude relative risk. [¤]aRR – relative risk adjusted for maternal body mass index (weight in kilograms divided by the square of height in meters), age in years, ART regimen, viral suppression, CD4 count, education, and household food insecurity.

Supplemental Table 2: Cofactors of growth faltering (becoming underweight, wasted, or stunted) during follow-up in the overall cohort of HEU infants in analysis to 48 weeks postpartum

Variables	Underweight[s]			Stunting[¶]			Wasting		
	**cRR[¥] (95% CI)**	**aRR[¤] (95% CI)**	**P-value**	**cRR[¥] (95% CI)**	**aRR[¤] (95% CI)**	**P-value**	**cRR[¥] (95% CI)**	**aRR[¤] (95% CI)**	**P-value**
IPT started during pregnancy	1·28 (0·97, 1·70)	**1·36 (1·04, 1·78)**	**0·027**	1·12 (0·94, 1·34)	1·16 (0·97, 1·39)	0·10	0·98 (0·75, 1·29)	0·98 (0·75, 1·27)	0·86
Male infant	1·37 (1·03, 1·82)	**1·40 (1·07, 1·85)**	**0·016**	1·23 (1·03, 1·48)	**1·23 (1·03, 1·48)**	**0·022**	0·81 (0·61, 1·06)	0·83 (0·63, 1·09)	0·18
Mother's BMI[§]	0·91 (0·89, 0·94)	**0·91 (0·88, 0·94)**	**<0·0001**	0·98 (0·96, 1·00)	**0·98 (0·96, 1·00)**	**0·041**	0·93 (0·90, 0·96)	**0·92 (0·89, 1·09)**	**<0·0001**
Mother's age in years	1·00 (0·97, 1·02)	1·00 (0·98, 1·03)	0·77	0·99 (0·97, 1·00)	0·99 (0·97, 1·00)	0·15	1·03 (1·00, 1·05)	**1·03 (1·01, 1·06)**	**0·010**
ART regimen			0·054			0·013			0·60
3TC/FTC,TDF,EFV	1	1		1	1				
3TC, ZDV, EFV	0·94 (0·25, 3·48)	0·99 (0·28, 3·49)		1·47 (0·75, 2·88)	1·44 (0·73, 2·84)		-	-	
3TC/FTC, LVP/ATV, TDF/ZDV	0·76 (0·25, 2·32)	0·65 (0·22, 1·90)		1·43 (0·85, 2·42)	1·34 (0·79, 2·26)		0·84 (0·30, 2·31)	0·78 (0·29, 2·15)	
3TC/FTC, ZDV/TDF, NVP	1·35 (0·92, 1·96)	**1·66 (1·14, 2·43)**		1·34 (1·05, 1·71)	**1·48 (1·15, 1·91)**		1·15 (0·79, 1·68)	1·14 (0·78, 1·69)	
Viral load (≥40 HIV copies /ml)	1·26 (0·95, 1·67)	1·27 (0·95, 1·71)	0·11	1·09 (0·91, 1·31)	1·18 (0·97, 1·44)	0·089	0·94 (0·70, 1·24)	0·94 (0·70, 1·27)	0·69
CD4 count (cells/mm^3)			0·20			0·20			0·60
<200	1	1		1	1		1	1	
200-500	0·68 (0·41, 1·14)	0·64 (0·39, 1·06)		1·12 (0·97, 1·30)	0·94 (0·64, 1·37)		1·19 (0·65, 2·19)	1·22 (0·66, 2·23)	
>500	0·72 (0·43, 1·19)	0·72 (0·44, 1·21)		1·00 (1.00, 1·00)	1·10 (0·75, 1·62)		1·16 (0·63, 2·13)	1·25 (0·68, 2·32)	
Mother's education			0·40			0·079			0·092
Primary school completed or less	1	1		1	1	1	1	1	
Secondary school	0·91 (0·66, 1·25)	0·89 (0·65, 1·22)		0·78 (0·64, 0·94)	**0·80 (0·65, 0·97)**		1·46 (1·03, 2·06)	**1·44 (1·02, 2·04)**	
Some college education	0·63 (0·33, 1·23)	0·65 (0·34, 1·24)		0·81 (0·56, 1·17)	0·80 (0·55, 1·16)		1·10 (0·59, 2·04)	1·17 (0·63, 2·18)	
Food insecure household	0·82 (0·52, 1·30)	0·77 (0·49, 1·20)	0·25	0·99 (0·75, 1·30)	0·94 (0·72, 1·24)	0·67	0·84 (0·54, 1·30)	0·83 (0·53, 1·30)	0·41

[s]Underweight – defined as weight-for-age (WAZ)<-2). [¶]Wasting – defined as weight-for-length (WLZ)<-2. [β]Stunting – defined as length-for-age (LAZ)<-2.[¥]cRR – crude relative risk. [¤]aRR – relative risk adjusted for maternal body mass index (weight in kilograms divided by the square of height in meters), age in years, ART regimen, viral suppression, CD4 count, education, and household food insecurity. [§]Body mass index is the weight in kilograms divided by the square of height in meters.
