# Supplementary material for: Effect of pregnancy versus postpartum maternal isoniazid preventive therapy on infant growth in HIV-exposed uninfected infants: a post-hoc analysis of the TB APPRISE trial
Source: eClinicalMedicine. 2023 Mar 17;58:101912. doi: 10.1016/j.eclinm.2023.101912 (PMC10031034; doi:10.1016/j.eclinm.2023.101912)
Supplement: IMPAACT P1078 TB APPRISE Study Team [file mmc2.docx]

| **First Name** | **Surname** |
| --- | --- |
| Timothy R. | Sterling |
| Renee | Browning |
| Katie | McCarthy |
| Lisa | Aaron |
| Katherine | Shin |
| Amanda | Golner |
| Bonnie | Zimmer |
| Jyoti S. | Mathad |
| Savita | Pahwa |
| Vandana | Kulkarni |
| Diane | Costello |
| Vivian | Rexroad |
| Monica | Gandhi |
| Joan | Du Plessis |
| Amy | James Loftis |
